# Study of Structural and Strength Changes in Lithium-Containing Ceramics—Potential Blanket Materials for Nuclear Power, Subjected to High-Dose Proton Irradiation

**DOI:** 10.3390/ma15165572

**Published:** 2022-08-13

**Authors:** Askhat Berguzinov, Artem L. Kozlovskiy, Ainagul A. Khametova, Dmitriy I. Shlimas

**Affiliations:** 1 Department of Heat Power Engineering, Toraighyrov University, Pavlodar 140000, Kazakhstan; 2 Laboratory of Solid State Physics, The Institute of Nuclear Physics, Almaty 050032, Kazakhstan; 3 Engineering Profile Laboratory, L.N. Gumilyov Eurasian National University, Nur-Sultan 010008, Kazakhstan

**Keywords:** materials for nuclear power, blanket, Li_2_TiO_3_ ceramic, swelling, radiation defects

## Abstract

The paper considers the hydrogenation processes in Li_2_TiO_3_ ceramics under irradiation with protons with an energy of 500 keV and fluences of 1 × 10^10^–5 × 10^17^ ion/cm^2^. The choice of the type of irradiation, as well as the irradiation fluences, is based on the possibilities of modeling hydrogenation processes and studying the kinetics of structural changes caused by the accumulation of radiation damage. The choice of Li_2_TiO_3_ ceramics as objects of research is due to their prospects for using as blanket materials of thermonuclear reactors for the tritium production and accumulation. It was found that the formation of point defects and their subsequent evolution associated with the formation of complex compounds and the filling of pores, followed by the formation of gas-filled bubbles, the presence of which leads to a decrease in crack resistance and resistance to destruction of the near-surface layer. Based on the data on structural changes and evolution of the crystal lattice parameters, its swelling, a description of the destruction processes associated with hydrogenation in Li_2_TiO_3_ ceramics was proposed. Also, during the studies, it was found that at irradiation fluences above 1 × 10^17^ ion/cm^2^, the appearance of impurity inclusions characteristic of the TiO_2_ phase was observed, the presence of which indicates the crystal lattice destruction processes because of accumulation of radiation damage and deformations caused by them. Critical doses are established at which there is a sharp deterioration in strength and crack resistance, reflecting the resistance of ceramics to mechanical external influences.

## 1. Introduction

For the successful development of thermonuclear energy, which is the key to solving the energy crisis in the near future, it is necessary to have fuel to maintain thermonuclear reactions [1,2,3]. One such fuel is tritium, which unlike classical nuclear fuels (uranium and plutonium) has a shorter half-life and eliminates the accumulation of radioactive waste, making it environmentally safer. Also, much more energy is released in nuclear reactions with tritium, which can subsequently be transformed into thermal energy [4,5]. However, despite all the advantages of using tritium fuel, there is one big problem that needs to be addressed immediately. This problem consists in the low natural tritium content, and the currently known methods for producing tritium cannot cover all the needs for it in the case of a full-fledged launch of technologies for creating thermonuclear reactors [6,7,8].

One of the solutions to this problem is the production of tritium in the fusion reactor itself during its operation, which will make it possible to maintain the reactor’s operability for a long time [9,10]. For this, it is proposed to use blankets containing lithium, which, as is known during a nuclear reaction with neutrons, decays into helium and tritium, and this reaction is accompanied by the release of a large amount of energy [11,12,13,14,15]. The use of lithium-containing ceramics as blanket materials for tritium production is one of the most promising solutions to the problem of tritium in thermonuclear power engineering [16,17].

In this regard, in the last few years, much attention has been paid to the study of methods for obtaining lithium-containing ceramics to search for optimal ceramic types, the combination of properties of which will allow for solving several problems associated with the tritium production [18,19,20,21,22]. One of these problems is the radiation damage accumulation, as well as gas swelling and embrittlement associated with the occurrence of nuclear reactions and subsequent interaction with the products of these reactions [23,24,25]. From the point of view of structural changes caused by their accumulation, the most dangerous are helium and hydrogen, which, due to their nature and low solubility, are capable of accumulating in the structure of ceramics. The accumulation of reaction products, as well as the probable filling of pores by them, the presence of which can be caused both by the synthesis processes and tritium burnout, can lead to the destruction of ceramics, deterioration of their properties, decrease in mechanical stress and crack resistance. Such consequences can have a negative effect on the service life of lithium-containing ceramics, as well as reduce the tritium production efficiency [26,27,28,29]. In this connection, in recent years, great interest has been paid to experimental work related to the study of the radiation resistance of lithium-containing ceramics in the processes of hydrogenation, gas swelling, or helium embrittlement, the results of which will provide a more complete picture of the mechanisms and processes occurring in ceramics during radiation damage.

Based on the foregoing, the aim of this work is to study the processes of hydrogenation and subsequent gaseous swelling in Li_2_TiO_3_ ceramics under proton irradiation with doses of 1 × 10^10^–5 × 10^17^ ion/cm^2^. The results of this study will make it possible to determine the resistance of the selected ceramics to structural and strength degradation depending on the radiation damage degree.

## 2. Experimental Part

The samples were irradiated with protons with an energy of 500 keV and fluences of 1 × 10^10^–5 × 10^17^ ion/cm^2^. The samples were irradiated at the UKP-2-1 accelerator located at the Institute of Nuclear Physics (Almaty, Kazakhstan). Irradiation of the studied samples was carried out in a vacuum at a temperature of 300 K (room temperature), the samples were placed on special water-cooled target holders in order to prevent overheating of the samples during irradiation. Such a wide range of irradiation fluences made it possible to evaluate changes in the structural and strength parameters of ceramics depending on the density of accumulated defects and their evolution. The choice of irradiation conditions, in particular, irradiation fluences, is based on the possibilities of modeling the evolution of point and dislocation defects in the structure of the damaged layer from the conditions for the formation of single isolated defects to the case of deep overlap and the formation of defect compounds.

**Figure 1:**
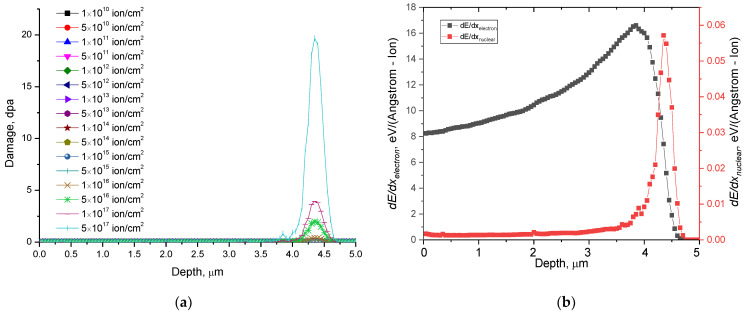
(**a**) Results of the calculation of the atomic displacement value from the depth of the passage of protons in the structure and the formation of the maximum of atomic displacements; (**b**) Dependence of the change in energy losses depending on the depth of penetration of incident particles in ceramics.

The assessment of the maximum proton path length in the material, as well as the accumulation of atomic displacements because of an increase in the irradiation fluence, was carried out by computer simulation in the SRIM Pro 2013 program code, which makes it possible to determine the nature of the interaction of incident protons with the crystal structure of ceramics using the detailed Kinchin–Pease model (detailed KP). The simulation was performed using a model that takes into account damage cascades. The Displacement Energy values for Li and Ti atoms were chosen to be 25 eV, for O atoms 28 eV. Figure 1a shows the results of calculations of the change in the atomic displacement value depending on the irradiation fluence. The value of atomic displacements was calculated using Formula (1) taken from the work of G.W. Egeland et. al. [30].

(1) $$\mathrm{dpa}=\left(\frac{10^{8}\times \mathrm{fluence}}{8.38\times 10^{22}}\right)\times (\mathrm{Vacancy}.\mathrm{txt}),$$

Vacancy.txt is a calculation file taken from the results of modeling the processes of radiation damage and the depth of travel using the SRIM Pro 2013 program code. According to the results of modeling the interaction of protons with the energy of 500 keV using the SRIM Pro 2013 program code, the energy losses during interaction with the electron subsystem and the nuclear subsystem are dE/dx_electron_~15–17 keV/µm, dE/dx_nuclear_ ~ 0.07 keV/µm, respectively, the difference between the values is more than 1000 times. Such a strong difference in energy losses indicates that the greatest contribution to the change in the structural properties of ceramics along the entire trajectory of protons in a substance is made by ionization processes associated with a change in electron density during the interaction of incident particles with electron shells. Figure 1b shows the dependences of the change in energy losses along the trajectory of the incident particles in ceramics.

According to the data obtained, the maximum value of the proton free path in ceramics is no more than 4.3–4.5 μm. At a given proton penetration depth, there is a maximum displacement of atoms from the equilibrium position, because during the interaction of incident protons with matter, the distribution of energy losses is such that the maximum nuclear losses occur at the maximum penetration depth, while electronic losses are distributed almost uniformly along the entire trajectory of the incident particles. According to estimated calculations of the atomic displacement values, the most significant values are observed for fluences of more than 10^15^ ion/cm^2^, which are characterized by the radiation damage accumulation effects due to the formation of defect overlap regions. At fluences above 10^15^ ion/cm^2^, the value of atomic displacements exceeds 0.01 dpa, and a further increase in the irradiation fluence leads to its increase by several orders of magnitude. Such a change in the magnitude of displacements indicates the accumulation of radiation damage, the formation of defective inclusions and regions of disorder. The maximum atomic displacement value is 19–20 dpa at a fluence of 5 × 10^17^ ion/cm^2^.

X-ray phase analysis and analysis of the studied ceramics before and after irradiation was carried out in the Bragg–Brentano geometry in the angular range of 2θ = 25–85°, fully reflecting the main changes in the diffraction pattern of the samples. X-ray diffraction patterns were taken using a D8 Advance ECO X-ray diffractometer (Bruker, Rheinstetten, Germany). The structural parameters were determined using the Diffrac EVA v.4.2 software code, which is used to refine the change in the position of diffraction lines, as well as the crystal lattice parameters and the degree of crystallinity of the samples under study.

The strength properties were determined using indentation methods implemented on a LECO microhardness tester with an indenter load of 100 N. A Vickers pyramid was used as an indenter. Determination of crack resistance was carried out by the single compression method at a compression rate of 0.2 mm/min. In order to collect statistical data and determine the measurement error, as well as to establish the repeatability of the destruction mechanisms during the accumulation of radiation damage, mechanical tests were carried out on a series of samples of 10 pieces.

## 3. Results and Discussion

**Figure 2:**
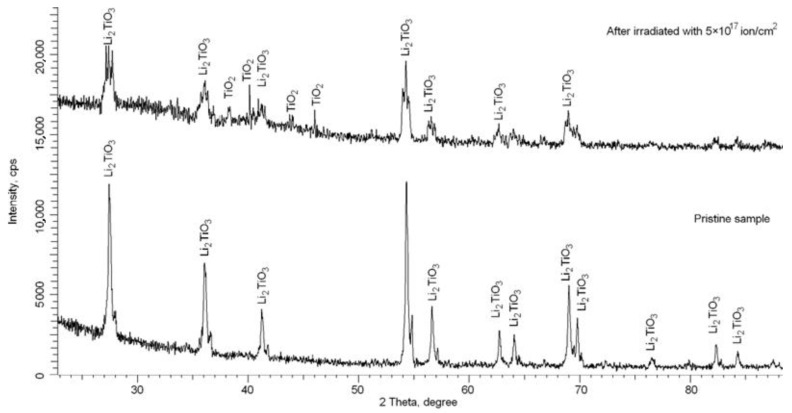
Results of X-ray phase analysis of the studied samples before and after irradiation with a fluence of 5 × 10^17^ ion/cm^2^.

Figure 2 shows the results of X-ray diffraction analysis of the studied samples in the initial state and irradiated with a fluence of 5 × 10^17^ ion/cm^2^, characterizing the structural and phase changes of the samples during irradiation. Intermediate diffraction patterns are not provided due to the absence of significant changes in diffraction patterns in the form of the appearance of additional peaks characteristic of the formation of impurity inclusions or phase transformations. The main changes are associated with a change in the intensity and position of diffraction reflections, which reflects the deformation processes and the degree of structural changes associated with irradiation.

In the initial state, the presented diffraction pattern indicates that the angular position, as well as the main diffraction maxima, corresponds to the Li_2_TiO_3_ phase with a monoclinic type of crystal structure, characterized by the parameters a = 5.0301 Å, b = 8.7871 Å, c = 9.7061 Å, β = 99.97°. At the same time, the shape of diffraction reflections, as well as their intensity, indicates a high structural ordering degree and a low content of the defect fraction, the presence of which is associated with the technological processes of ceramic synthesis, as well as grain sizes.

For irradiated samples with a maximum fluence (5 × 10^17^ ion/cm^2^), the shape of diffraction reflections indicates a strong distortion and deformation of the crystal structure caused by irradiation, as well as partial amorphization of the ceramic. Also, a detailed analysis of the obtained diffraction patterns revealed the presence of low-intensity diffraction reflections in the region of 2θ = 37–45°, characteristic of the TiO_2_ phase, and the intensity and width of the observed reflections indicate nano-sized inclusions (no more than 10–15 nm), moreover, strongly deformed and distorted (strong asymmetry of diffraction reflections characteristic of impurity inclusions).

An analysis of the shape and intensity of the main diffraction reflections characterizing the Li_2_TiO_3_ phase before and after irradiation showed that, after irradiation, the reflections have a strongly distorted asymmetric shape, which indicates crystal structure deformation, as well as disordering and amorphization processes resulting from the accumulation of radiation defects in the structure of ceramics. In this case, a decrease in intensity, as well as a change in the shape and width of diffraction reflections, indicates dimensional changes in crystallites under the action of irradiation, and, consequently, dislocation density. At the same time, an analysis of the ratio of the areas of reflections and halo, which characterizes the concentration of disorder regions, indicates amorphization of the structure of the samples during irradiation.

**Figure 3:**
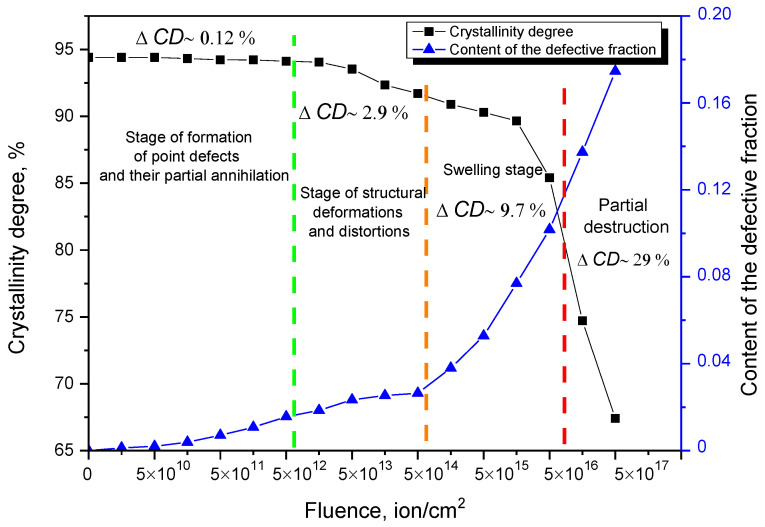
Results of the change in the crystallinity degree and the concentration of the defective fraction: dotted lines indicate the boundaries of areas characteristic of various stages of structural changes associated with deformation processes caused by irradiation (These changes in the degree of crystallinity were obtained by analyzing X-ray diffraction patterns, the concentration of the defective fraction was estimated using Formula (2)).

Figure 3 shows the results of change in the crystallinity degree and the concentration of the defective fraction (defective inclusions), the presence of which is due to a change in the number of defects in the structure, their accumulation and further evolution.

Data were obtained from observed changes using X-ray diffraction and subsequent analysis of structural changes. The degree of crystallinity is a value that characterizes the degree of structure ordering, which is estimated from the ratio of the areas of diffraction reflections and the halo area, which is characteristic of disordered inclusions in the structure.

The swelling was estimated from the change in the volume of the crystal lattice, which characterizes the integral change in the volume of the crystal structure subjected to irradiation. The value of the defective fraction was estimated using Formula (2).

(2) $$F_{d}=1-\frac{V_{0}}{V},$$

where *V*_0_, *V* are the volume of the crystal lattice in the initial and irradiated states.

An analysis of the change in these quantities makes it possible to evaluate the integral changes in the crystal structure associated with the formation of point defects, their accumulation as a result of an increase in the irradiation fluence, and subsequent evolution, which is expressed in the swelling of the crystal lattice as a result of its deformation (the region characteristic of irradiation fluences of 10^15^–10^16^ ion/cm^2^) and subsequent destruction of the irradiated material at high irradiation fluences, which are characterized by a large number of formed defective inclusions, as well as atomic displacements, leading to the appearance of strong distortions and overstressed defective or disordered regions.

The change in these values depending on the irradiation fluence characterizes evolution of the stability of the damaged layer of ceramics during the formation and accumulation of point defects, the formation of compounds by them, as well as regions of disorder. At low irradiation fluences, it was found that the crystallinity degree practically does not change with increasing fluence, which indicates that the processes of structure disordering or the formation of amorphous-like inclusions are not initiated. At the same time, the change in the concentration of point defects or a defective fraction for irradiation fluences of 5 × 10^10^–5 × 10^12^ ion/cm^2^ is no more than 2% of the initial value, which indicates that at these irradiation fluences, isolated point defects are formed, which have little effect on structural changes. For irradiation fluences of 5 × 10^13^–5 × 10^14^ ion/cm^2^, equiprobable changes in the crystallinity degree and concentration of the defective fraction are observed, which indicates that an increase in the irradiation fluence leads both to an increase in the concentration of formed point defects and the formation of highly disordered regions by them.

These effects can be quite well explained by the following facts. Firstly, an increase in the irradiation fluence leads to a decrease in the distance between damaged regions formed along the trajectory of charged particles in the material, which leads to a decrease in the concentration of isolated point defects, as well as an increase in the probability of the formation of more complex defect compounds. In turn, with an increase in the irradiation fluence, the effect of the possible annihilation of point defects in the case of their isolation sharply decreases, which leads to the accumulation of defects in the structure of the damaged layer and its deformation, which are expressed in the form of distortions of the crystal lattice and an increase in its volume. Also, with an increase in the irradiation fluence, the concentration of implanted hydrogen increases, which, due to its high mobility, can migrate over the structure and, by forming complex defects with vacancies, fill pores and voids, thereby forming gas-filled bubbles. The formation of bubbles also has a negative effect on the swelling and resistance of the material to deformation and disorder. In this case, the stability of the structure to disordering, the factors of amorphization associated with the destruction of crystalline bonds, and the formation of defective regions, with an increase in the concentration of point defects, are equally probable. Thus, it can be concluded that both amorphization and an increase in the defective fraction have the same effect on the change in structural characteristics at irradiation fluences of 5 × 10^13^–5 × 10^14^ ion/cm^2^.

At irradiation fluences of 10^16^–10^17^ ion/cm^2^, a sharp increase in the defective fraction is observed, which is due to the swelling effects associated with the implanted hydrogen accumulation, as well as an increase in the atomic displacement value. At the same time, the change in the crystallinity degree has the same downward trend as with fluences of 10^13^–10^15^ ion/cm^2^, which indicates that the accumulation of amorphous inclusions or disorder areas because of irradiation occur with equal probability. However, at fluences of 5 × 10^16^–10^17^ ion/cm^2^, a sharp deterioration in the crystallinity degree is observed, which may be due to the processes of partial destruction of the crystal structure and the formation of impurity inclusions. At the maximum irradiation fluence of 5 × 10^17^ ion/cm^2^, the crystallinity degree decreases by more than 25–30%, which indicates rather strong structural changes and amorphization of the damaged layer.

**Figure 4:**
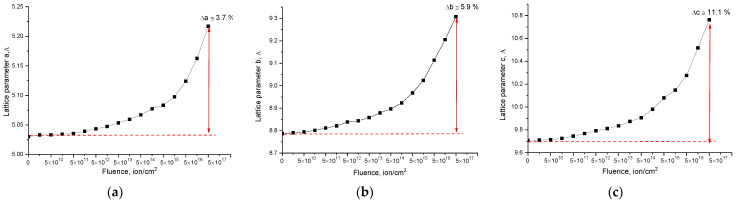
Results of change in the crystal lattice parameters: (**a**) parameter *a*; (**b**) parameter *b*; (**c**) parameter *c*.

Figure 4 shows the dose dependences of the change in the crystal lattice parameters of the studied samples of Li_2_TiO_3_ ceramics with a monoclinic type of structure. The general form of the changes is indicative of tensile deformation processes associated with the effects of irradiation and the formation of point defects in the structure of ceramics, as well as their further evolution during accumulation. The nature of parameter changes for all axes is the same, but the degree of change in values is different, which indicates anisotropic effects of stretching and deformation of the crystal structure.

The general appearance of the dependence can be divided into three characteristic stages, which have a pronounced dependence on the irradiation fluence, and the general dependences of changes can be described by an exponential law.

The first stage of changes in the parameters of the crystal lattice is typical for irradiation fluences of 5 × 10^10^–5 × 10^14^ ion/cm^2^, and the nature of the change is almost minimal and does not exceed 1–3%. Such small changes are because these fluences are characterized by the formation of single point defects isolated from each other, most of which annihilate in very short time intervals (10^−13^ s). In the case of isolation of single point defects, the formation of cluster or more complex defect compounds is impossible, and therefore structural changes are local in nature and do not have a great effect on the properties of ceramics. However, an increase in the irradiation fluence, and therefore, the effects of the displacement of atoms from the equilibrium position due to the accumulation of surviving point defects, leads to an increase in structural distortions and deformations in the crystal lattice. In the case of an increase in the irradiation fluence above 5 × 10^14^ ion/cm^2^, a nonlinear increase in the deformation of the crystal lattice is observed due to the broadening of the parameters. It is also worth noting that small changes in the crystal lattice parameters at irradiation fluences of 10^11^–10^13^ ion/cm^2^ can be due to effects similar to the effects of thermal expansion of the crystal lattice, due to the creation of local point defects, as well as the conversion of the kinetic energy of incident particles into thermal energy during collisions and interactions with the crystal lattice. In this case, an increase in the irradiation fluence leads to the fact that this effect is leveled or suppressed by the deformation and distorting effects of the crystal structure.

At an irradiation fluence above 10^13^ ion/cm^2^, a sharp increase in the changes in the crystal lattice parameters towards an increase is observed, while the nature of the changes in the parameters is close to exponential. In this case, the general form of changes in the parameters of the crystal lattice indicates an anisotropic distortion of the crystal lattice along the axes. An analysis of the maximum changes in the values of the crystal lattice parameters at the maximum irradiation fluence indicates that the anisotropic distortion is most pronounced along the b and c axes (these changes are shown in Figure 4).

**Figure 5:**
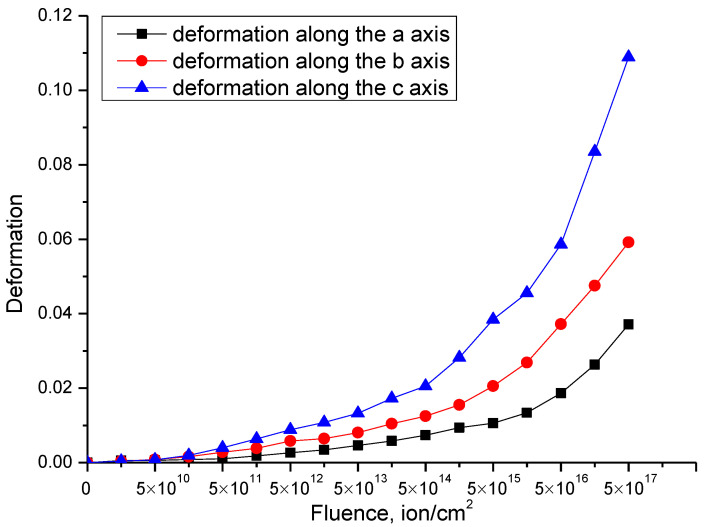
Deformation of the crystal lattice of Li_2_TiO_3_ ceramics depending on the irradiation fluence.

Figure 5 shows the results of the evaluation of the lattice deformation along the axes *a, b, c*, calculated based on changes in the parameter values presented in Figure 4. The general appearance of the change in the crystal lattice deformation depending on the irradiation fluence indicates tensile deformation or swelling of the crystal lattice. At the same time, changes in the crystal lattice deformation are also two-stage in nature and are close to the exponential description depending on the irradiation fluence. It should also be noted that the value of changes in the crystal lattice deformation along different axes is different, which indicates that the crystal lattice deformation, depending on the irradiation fluence, and, consequently, the accumulated dose of structural damage, has an anisotropic nature.

It can be seen from the presented data that the main differences in the crystal lattice deformation and an increase in the anisotropic nature of the crystal lattice deformation were previously found for SiC ceramics subjected to irradiation with helium ions [31]. In this case, the anisotropic deformation of the crystal lattice of SiC was explained by the authors by the hexagonal type of structure, as well as the difference in chemical and crystalline bonds of the Si-C, Si-Si, C-C type, the partial destruction of which can lead to the crystal lattice destruction at high irradiation fluences, and the accumulation of implanted hydrogen, followed by the formation of gas-filled bubbles. In the case of lithium-containing ceramics, with a monoclinic type of crystal structure, and many variations in chemical bonds, the anisotropic deformation processes can be more pronounced due to the structure properties. In this case, as was established from the X-ray diffraction data, at high irradiation fluences above 10^17^ ion/cm^2^, the presence of TiO_2_ impurity phases was found in the ceramic structure, the formation of which can be explained by a strong deformation of the crystal lattice, followed by the breaking of chemical and crystalline bonds, as well as the radiation damage accumulation and swelling.

**Figure 6:**
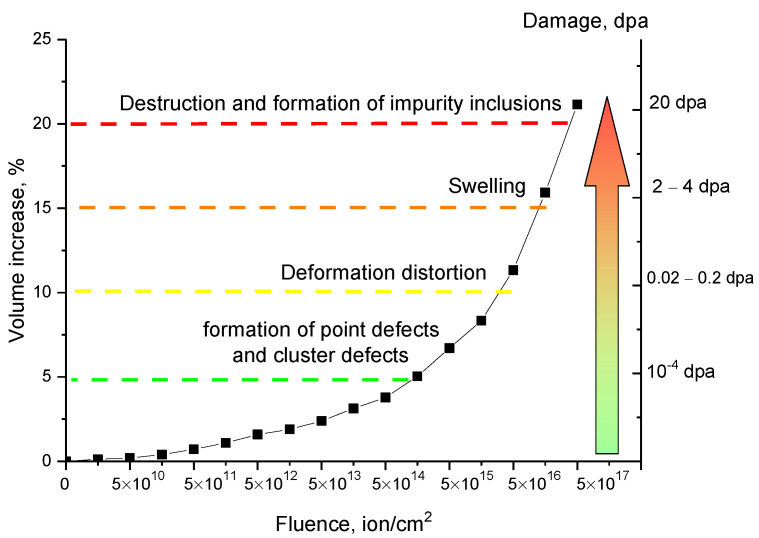
Dynamics of swelling of the crystal lattice volume depending on the fluence.

Figure 6 shows the results of changes in the crystal lattice volume of the studied ceramics depending on the irradiation fluence and, consequently, the accumulated radiation damage. The general trend in the change in the Li_2_TiO_3_ ceramic crystal lattice volume depending on the irradiation fluence corresponds to the observed effects of crystal lattice deformation and is because of swelling and subsequent destruction at high irradiation fluences. The trend of change in the crystal lattice volume can be divided into four characteristic areas, which are characterized by structural effects, their evolution, and the crystal lattice swelling degree.

The first stage is characterized by a low swelling degree (no more than 3–5%) and is typical for irradiation fluences of 5 × 10^10^–5 × 10^14^ ion/cm^2^. At the same time, the trend of change in the swelling value depending on the irradiation fluence is close to linear, and the changes with an increase in the irradiation fluence are quite small (no more than 0.3–0.4% with an increase in the irradiation fluence by an order of magnitude). Such small changes in the swelling degree are due to the low degree of radiation damage, most of which, at low radiation fluences, annihilate with each other or with defects formed during synthesis. It should be considered that at low irradiation fluences (5 × 10^10^–5 × 10^12^ ion/cm^2^), most of the radiation-induced defects are isolated from each other, which excludes the possibility of the formation of complex defects or regions with a high structural distortion degree.

The second stage is typical for irradiation fluences of 10^15^–10^16^ ions/cm^2^ and is characterized by a sharp increase in the crystal lattice swelling degree from 5 to 10–12%. At this stage, as was shown earlier, anisotropic distortions of the crystal lattice, associated with partial breaking of crystal and chemical bonds, begin to play an important role. In this case, as shown in Figure 3, these irradiation fluences are also characterized by the initiation of amorphization processes associated with the radiation damage accumulation and a decrease in the structural ordering degree.

The third stage is typical for the formation processes of gas-filled bubbles during the accumulation of implanted hydrogen in structurally distorted or deformed cavities, which leads to uncontrolled swelling of the crystal lattice volume.

The fourth stage consists in partial amorphization of the structure due to strong deformation of the crystal lattice and the formation of impurity inclusions of the TiO_2_ type, the presence of which indicates the processes of polymorphic transformations or the processes of complete destruction of Li_2_TiO_3_ grains.

Analyzing the dependences of structural quantities, we can draw the following conclusions about the mechanisms that occur during the accumulation of radiation damage in the structure. At low irradiation fluences of 10^10^–10^12^ ion/cm^2^, the main changes in the structural properties are due to the formation of isolated point defects, most of which annihilate with each other or during interaction with structural defects. At the same time, distortions of the crystal lattice, as well as its deformation and increase in volume in this case are minimal, and the crystallinity degree remains within the permissible error. The nature of structural changes for these irradiation fluences is isotropic. At irradiation fluences of 10^13^–10^15^ ion/cm^2^, an anisotropic deformation of the crystal lattice occurs, as well as an increase in the concentration of defective inclusions and the formation of amorphous or highly disordered regions in the damaged layer structure. Anisotropic distortion of the crystal lattice, in turn, leads to an increase in its volume, accompanied by swelling effects. However, a sufficiently high degree of resistance to structural changes at given irradiation fluences can also be due to a rather high dislocation density and small grain sizes, which leads to partial inhibition of hydrogen migration and its subsequent agglomeration, leading to the formation of gas-filled cavities. In this case, the main contribution to the destruction of the damaged layer is made by the processes associated with the cumulative effect of the defective fraction and anisotropic distortion of the crystal lattice.

At irradiation fluences above 10^15^ ion/cm^2^, the main contribution to the change in the structural properties is made by the effects associated with crystal lattice swelling and partial amorphization of the damaged layer, including those caused by the formation of TiO_2_ impurity inclusions in the composition of the damaged layer. Also, with an increase in the irradiation fluence, an increase in the anisotropic distortion of the crystal lattice is observed, which contributes to its destruction with the subsequent formation of impurity inclusions, the appearance of which leads to a sharp decrease in the structural ordering degree.

**Figure 7:**
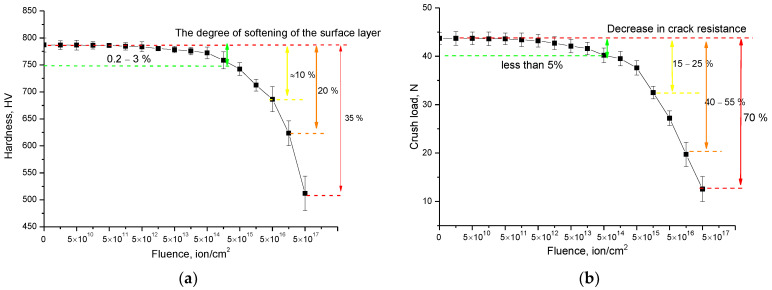
(**a**) Results of changes in the hardness of the near-surface layer depending on the irradiation fluence; (**b**) Results of the change in the crack resistance of the near-surface layer depending on the irradiation fluence.

Determination of the strength properties of ceramics and the dynamics of their change depending on the irradiation fluence and accumulated radiation damage has a significant role in studying the applicability of ceramics and their resistance to radiation. Figure 7 shows the data on changes in the hardness and crack resistance values, which make it possible to evaluate the strength characteristics of ceramics.

The general view of the trend in strength characteristics has two distinct sections, described by different trend lines. The first section is characterized by small changes in strength characteristics, which indicates a sufficiently high stability and resistance of ceramics to external influences. This area is typical for irradiation fluences of 10^10^–5 × 10^14^ ion/cm^2^, and the values of changes in hardness and crack resistance were less than 5%. Such small changes in strength properties in this fluence range can be explained by the following factors. First, at irradiation fluences of 10^10^–10^12^ ion/cm^2^, most of the radiation-induced structural damage, according to X-ray diffraction data, is isolated and does not make a large contribution to the change in the resistance of the material to radiation exposure. Also, as is known, at low irradiation fluences, a part of isolated point defects can annihilate, which also affects the degree of radiation damage. A slight decrease in strength properties at irradiation fluences above 5 × 10^13^ ion/cm^2^ is due to the cumulative effect associated with the formation of defect overlap regions with an increase in irradiation fluence, as well as the formation of defective inclusions in the damaged layer structure. Also, such small changes can be associated with dislocation hardening due to the small size of crystallites from which ceramics are formed. A large dislocation density near the grain boundaries can lead to a decrease in the rate of nucleation and propagation of microcracks in the structure under external influences, which leads to hardening and an increase in crack resistance.

The second stage of the change in strength properties is associated with a sharp deterioration in hardness and crack resistance with an increase in the irradiation fluence. These changes can be related to the fact that at given irradiation fluences, the main structural changes are due to partial amorphization processes and an increase in the concentration of the defective fraction due to an increase in structural disordering and atomic displacements. At the same time, according to the assessment of the values of the decrease in hardness and crack resistance, it was found that an increase in the irradiation fluence above 5 × 10^15^ ion/cm^2^ leads to a greater decrease in the crack resistance (by more than two times in comparison with the results of changing the hardness value). This behavior of the strength characteristics can be explained by the effects of strong disordering of the structure, as well as deformation effects associated with anisotropic distortion of the structure and the formation of impurity inclusions.

**Figure 8:**
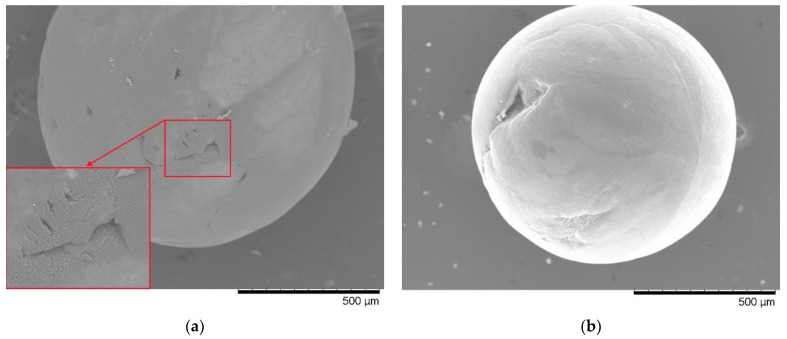

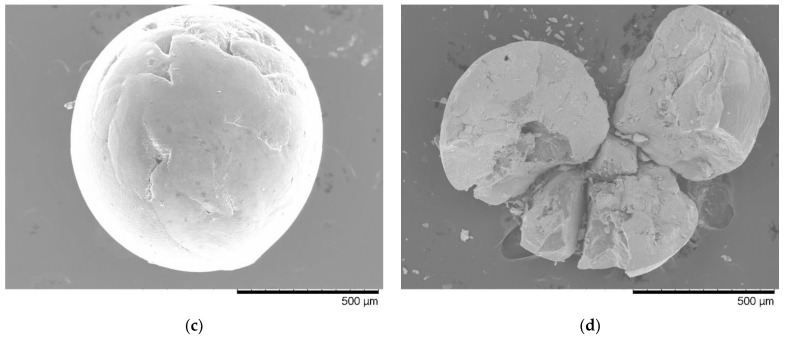
Results of morphological studies of the observed samples after mechanical tests: (**a**) initial sample; (**b**) irradiation with a dose of 1 × 10^13^ ion/cm^2^; (**c**) irradiation with a dose of 1 × 10^16^ ion/cm^2^; (**d**) irradiation with a dose of 5 × 10^17^ ion/cm^2^.

Figure 8 shows the results of morphological changes in Li_2_TiO_3_ ceramics after irradiation with various irradiation fluences and mechanical impact with an indenter when measuring hardness. SEM data—images are presented as illustrative examples demonstrating the resistance of ceramics to indentation at an indenter pressure of 100 N. As can be seen from the data presented, when the initial sample is exposed to the indenter, the trace from the indenter is insignificant, and also has an irregular asymmetric shape, the presence of which indicates a high resistance to external influences. It is also worth noting that, under the influence of the indenter, the formation of longitudinal microcracks is observed near the indentation.

For samples irradiated with a dose of 10^13^ ion/cm^2^ during indentation, a slight increase in the indenter print is observed, which indicates deterioration in the strength properties and hardness of the near-surface layer subjected to irradiation. In this case, the formation of branched microcracks is observed near the indenter imprint.

More serious changes are observed for samples irradiated with a fluence of 10^16^ ion/cm^2^, which consist in the formation of cracks and cleavages during indentation, the presence of which is associated with a deterioration in strength properties, as well as the accumulation of deformation distortions and a high density of dislocation defects, which has a negative effect on the resistance of ceramics to external influences.

At the maximum irradiation fluence of 5 × 10^17^ ion/cm^2^, indentation leads to the complete destruction of ceramics, which is due to the high concentration of accumulated defects, as well as atomic displacements and implanted hydrogen, leading to swelling and destruction of the damaged irradiated layer.

Thus, the obtained dependences of the change in strength indicate that the accumulation of deformation distortions and amorphous inclusions leads to a decrease in the resistance of ceramics to cracking and a deterioration in strength properties. Analyzing the results of the strength characteristics of ceramics, it can be concluded that irradiation fluences of 10^16^–5 × 10^17^ ion/cm^2^ are critical doses of proton irradiation (more than 5 dpa) for synthesized Li_2_TiO_3_ ceramics, leading to accelerated degradation of the damaged near-surface layer of ceramics, accompanied by its partial destruction.

## 4. Conclusions

The paper presents the main results of studies of the proton irradiation effect on the structural and strength properties of synthesized Li_2_TiO_3_ ceramics, which have great prospects as blanket materials for tritium production. Determination of structural changes was carried out by a comprehensive analysis of X-ray diffraction patterns. During the studies carried out, the mechanisms leading to structural and strength changes caused by the radiation damage accumulation in the structure of ceramics were established. With an increase in the irradiation fluence, it was found that the crystal lattice deformation has an anisotropic nature, due to both the accumulation of defects in the structure and swelling processes. It has been determined that at an irradiation dose above 10^17^ ion/cm^2^, the formation of impurity inclusions is observed, leading to accelerated amorphization of the damaged layer and a sharp deterioration in the strength properties and crack resistance.

Further research in this direction will be related to the study of the effect of irradiation temperature on the resistance of ceramics to hydrogenation and subsequent destruction. The interest in these studies is due to the need to obtain new data on the properties of lithium-containing ceramic materials to the effect of hydrogenation processes, which will allow us to evaluate the prospects for the use of these ceramics.

## Data Availability

Not applicable.

## References

[1] Zylstra A.B., Kritcher A.L., Hurricane O.A., Callahan D.A., Baker K., Braun T., Casey D.T., Clark D., Clark K., Döppner T. (2021). Record energetics for an inertial fusion implosion at NIF. Phys. Rev. Lett..

[2] Tajima T., Necas A., Mourou G., Gales S., Leroy M. (2021). Spent nuclear fuel incineration by fusion-driven liquid transmutator operated in real time by laser. Fusion Sci. Technol..

[3] Moses E.I., de la Rubia T.D., Storm E., Latkowski J.F., Farmer J.C., Abbott R.P., Kramer K.J., Peterson P.F., Shaw H.F., Lehman R.F. (2009). A sustainable nuclear fuel cycle based on laser inertial fusion energy. Fusion Sci. Technol..

[4] Kovari M., Coleman M., Cristescu I., Smith R. (2017). Tritium resources available for fusion reactors. Nucl. Fusion.

[5] Tanabe T. (2013). Tritium fuel cycle in ITER and DEMO: Issues in handling large amount of fuel. J. Nucl. Mater..

[6] Casey D.T., Frenje J.A., Johnson M.G., Manuel M.E., Rinderknecht H.G., Sinenian N., Séguin F.H., Li C.K., Petrasso R.D., Radha P.B. (2012). Evidence for stratification of deuterium-tritium fuel in inertial confinement fusion implosions. Phys. Rev. Lett..

[7] Xu R.H., Wen W., Zhao Y.K. (2022). Critical temperature for volume ignition of deuterium–tritium fuel in inertial confinement fusion: Effects of hydrodynamic instabilities. Phys. Plasmas.

[8] Pajuste E., Kizane G., Avotina L., Vitins A., Teimane A.S. (2021). Tritium retention in plasma facing materials of JET ITER-Like-Wall retrieved from the vacuum vessel in 2012 (ILW1), 2014 (ILW2) and 2016 (ILW3). Nucl. Mater. Energy.

[9] Tripathi B.M., Tyagi A.K., Prakash D. (2021). Synthesis and Processing of Li-Based Ceramic Tritium Breeder Materials. Handbook on Synthesis Strategies for Advanced Materials.

[10] Koga Y., Matsuura H., Katayama K., Otsuka T., Goto M., Hamamoto S., Ishitsuka E., Nakagawa S., Tobita K., Konishi S. (2022). Effect of nuclear heat caused by the 6Li (n, α) T reaction on tritium containment performance of tritium production module in High-Temperature Gas-Cooled reactor for fusion reactors. Nucl. Eng. Des..

[11] Eglitis R.I., Purans J., Gabrusenoks J., Popov A.I., Jia R. (2020). Comparative ab initio calculations of ReO_3_, SrZrO_3_, BaZrO_3_, PbZrO_3_ and CaZrO_3_ (001) surfaces. Crystals.

[12] Futamura Y., Kawamura H., Tsuchiya K. (2005). Data base for tritium solid breeding materials (Li_2_O, Li_2_TiO_3_, Li_2_ZrO_3_ and Li_4_SiO_4_) of fusion reactor blankets. Toyama Daigaku Suiso Doitai Kagaku Kenkyu Senta Kenkyu Hokoku.

[13] Shin-mura K., Otani Y., Ogawa S., Hoshino T., Sasaki K. (2017). Li vaporization properties of candidate materials for tritium breeder with high Li density. Fusion Eng. Des..

[14] Nakashima N., Beloglazov S., Hashimoto K., Nishikawa M. (2002). Isotope exchange reaction between gaseous hydrogen and tritium on Li_2_TiO_3_ grain surface. Fusion Sci. Technol..

[15] Kotomin E.A., Popov A.I., Stashans A. (1994). A novel model for F+ to F photoconversion in corundum crystals. J. Phys. Condens. Matter.

[16] Holmlid L. (2022). Muon-catalyzed fusion and annihilation energy generation will supersede non-sustainable T + D nuclear fusion. Energy Sustain. Soc..

[17] Taylor C.N. (2022). Hydrogen and its detection in fusion and fission nuclear materials—A review. J. Nucl. Mater..

[18] Popov A.I., Kotomin E.A., Kuklja M.M. (1996). Quantum chemical calculations of the electron center diffusion in MgO crystals. Phys. Status Solidi (B).

[19] Shin-mura K., Honda S., Hoshino T., Sasaki K. (2018). Li vaporization property of Li_8_ZrO_6_ and Li_5_AlO= as tritium breeders. Fusion Eng. Des..

[20] Futamura Y., Kawamura H., Tsuchiya K. (2001). Tritium breeding materials data base for fusion reactor blankets (4).(Li_2_O, Li_2_TiO_3_, Li_2_ZrO_3_ and Li_4_SiO_4_ solid breeding materials). Toyama Daigaku Suiso Doitai Kagaku Kenkyu Senta Kenkyu Hokoku.

[21] Kawagoe T., Nishikawa M., Baba A., Beloglazov S. (2001). Surface inventory of tritium on Li_2_TiO_3_. J. Nucl. Mater..

[22] Kanazawa T., Nishikawa M., Yamasaki H., Katayama K., Kashimura H., Hanada T., Fukada S. (2011). Study on tritium release behavior from Li_2_ZrO_3_. Fusion Sci. Technol..

[23] Kulsartov T., Kenzhina I., Tolenova A., Kenzhin Y., Shaimerdenov A., Nesterov Y., Gizatulin S., Chikhray Y., Gluchshenko A. (2021). Modeling of hydrogen isotopes release from lithium ceramics Li_2_TiO_3_ during in-situ experiments using vacuum extraction method. Fusion Eng. Des..

[24] Millers D., Grigorjeva L., Chernov S., Popov A., Lecoq P., Auffray E. (1997). The temperature dependence of scintillation parameters in PbWO4 crystals. Phys. Status Solidi (B).

[25] Gu S., Ji B., Qi Q., Wang J., Zhou H.S., Zhang Y., Luo G.N. (2021). Effects of He irradiation on the microstructure and mechanical performance of Li_2_TiO_3_. Nucl. Fusion.

[26] Ipponsugi A., Katayama K., Hoshino T. (2021). The influence of the long-term heating under H2 atmosphere on the tritium release behavior from the neutron-irradiated Li_2_TiO_3_. Fusion Eng. Des..

[27] Sahoo D.R., Chaudhuri P., Swaminathan N. (2021). A molecular dynamics study of displacement cascades and radiation induced amorphization in Li_2_TiO_3_. Comput. Mater. Sci..

[28] Liao Y., Wu H., Ran N., Liu J., Luo T. (2022). The possible negative state of deuterium in LiAlO_2_ irradiated by 3keV D_2_^+^ at higher temperature. J. Nucl. Mater..

[29] Guo H., Shi Y., Wang H., Chen R., Shi Q., Lu T. (2022). Fabrication of fine-grained Li_2_TiO_3_ ceramic pebbles with enhanced crush load by rolling method: Optimization of Li/Ti ratio and sintering procedure. J. Nucl. Mater..

[30] Egeland G.W., Valdez J.A., Maloy S.A., McClellan K.J., Sickafus K.E., Bond G.M. (2013). Heavy-ion irradiation defect accumulation in ZrN characterized by TEM, GIXRD, nanoindentation, and helium desorption. J. Nucl. Mater..

[31] Tynyshbayeva K.M., Kadyrzhanov K.K., Kozlovskiy A.L., Kuldeyev Y.I., Uglov V., Zdorovets M.V. (2022). Study of Helium Swelling and Embrittlement Mechanisms in SiC Ceramics. Crystals.

